# Outcomes of high-dose intensity-modulated radiotherapy alone with 1 cm planning target volume posterior margin for localized prostate cancer

**DOI:** 10.1186/1748-717X-8-285

**Published:** 2013-12-06

**Authors:** Rafael Gadia, Élton T Leite, Flavia G Gabrielli, Gustavo N Marta, Fernando F Arruda, Carlos V Abreu, Samir A Hanna, Cecilia K Haddad, João F Silva, Heloisa A Carvalho, Bernardo Garicochea

**Affiliations:** 1 Department of Radiation Oncology, Hospital Sírio Libanês, Rua Dona Adma Jafet, 91, Sao Paulo, Brazil; 2 Department of Radiation Oncology, Instituto do Câncer do Estado de São Paulo, Av. Dr. Arnaldo, 251, Sao Paulo, Brazil; 3 Department of Medical Oncology, Hospital Sírio Libanês, Rua Dona Adma Jafet, 91, Sao Paulo, Brazil

## Abstract

**Background:**

Clinically localized prostate cancer may be treated by different approaches of radiation therapy. The aim of this study was to report the results of disease control and toxicity in patients with clinically localized prostate cancer treated with high dose IMRT alone with 1 cm PTV posterior margin.

**Methods:**

From September 2001 to April 2008, 140 patients with localized prostate cancer were treated with definitive IMRT (dose ≥ 74 Gy) without hormone therapy. Outcomes were measured from the conclusion of radiotherapy. Biochemical failure was defined as PSA nadir + 2.0 ng/dL. Toxicities were assessed using the NCI-CTCAE-version 3.0. Median follow-up was 58 months.

**Results:**

Biochemical failure occurred in 13.6% of patients. Actuarial 5-year biochemical control rates were 91.7%, 82.5% and 85.9% for low-, intermediate-, and high-risk patients, respectively. Stage T2 patients presented a risk of biochemical failure almost three times higher than stage T1 (RR = 2.91; 95% CI: 1.04; 8.17). Distant metastases occurred in 3 (2%) patients. Five-year metastasis-free and overall survivals were 96% and 97.5%, respectively. Late grade 3 genitourinary and gastrointestinal toxicity rates were, respectively, 1.6% and 3%.

**Conclusion:**

High-dose IMRT alone with 1 cm posterior PTV margin was effective and safe for patients with localized prostate cancer.

## Background

The most common options for definitive treatment of early-stage prostate cancer are radical prostatectomy, brachytherapy, and external beam radiotherapy. In selected patients, androgen deprivation therapy or active surveillance may be used.

Radiotherapy (RT) with doses above 74 Gy is an important, well-established treatment for prostate cancer control. A meta-analysis of randomized clinical trials comparing high-dose versus low-dose RT for patients with clinically localized prostate cancer showed a significant reduction in biochemical failure over 5 years for the group of patients treated with high doses [1].

However, the delivery of high doses of radiation to the prostate is associated with higher rates of treatment-related gastrointestinal (GI) and genitourinary (GU) complications. Kuban et al. [2] showed that GI complication rates greater than grade 2 after 10 years of follow-up were twice as high (26% versus 13%) in patients treated to 78 Gy with three-dimensional conformal radiotherapy (3D-RT) than in those treated to 70 Gy with conventional RT.

In recent years, significant advances in conformal treatment delivery in the form of intensity-modulated radiotherapy (IMRT) have allowed excellent target coverage with high doses of radiation and concurrent dose reduction to adjacent normal tissues, thereby offering potentially more effective treatment. In a cohort of prostate cancer patients treated to 81 Gy with IMRT and a median follow-up of 99 months, the rate of grade ≥ 2 GI complications after 10 years was 3% [3].

It is worth emphasizing that the highly conformal nature of IMRT requires meticulous implementation and execution with rigorous quality control, in order to be effective.

The aim of this study was to report the results of disease control and toxicity in patients with clinically localized prostate cancer treated with definitive IMRT alone with 1 cm posterior planning target volume (PTV) margin and doses above 74 Gy.

## Methods

### Patients

Between September 2001 and April 2008, 634 consecutive patients with histologically proven prostate cancer were treated with IMRT at Sírio–Libanês Hospital in São Paulo, Brazil. Patients treated with doses < 74 Gy, subjected to any type of hormone therapy (HT), adjuvant or salvage RT, RT to pelvic lymph nodes, and patients with metastatic disease or lymph node involvement were excluded. Though, a total of 140 patients were included in the present study. All procedures were approved by the ethics committee at the Sírio-Libanês Hospital.

Risk groups were defined following the National Comprehensive Cancer Network Guidelines (NCCN) [4]. Patients were classified as follows: a) low risk: stage ≤ T2a and Gleason score ≤ 6 and PSA ≤ 10 ng/ml; b) high risk: stage ≥ T3 or Gleason score ≥ 8 or PSA > 20 ng/ml; c) intermediate risk: all other patients.

### Treatment

The treatment was carried out in daily fractions, five times per week, with an 18 MV linear accelerator.

Patients were simulated in a supine position with a support under the knees. All patients underwent a treatment planning computed tomography (CT) with a slice thickness of 3 mm, and the images were transferred to a treatment planning system. Patients were advised to have a comfortably full bladder and an empty rectum at the time of CT simulation.

The clinical target volume (CTV) for low-risk patients included only the prostate, while the CTV for high-risk patients included the prostate and the seminal vesicles, and the CTV for intermediate-risk patients included the prostate and the proximal portion of the seminal vesicles. The PTV consisted of the CTV plus a 10-mm margin in all directions, including the posterior, in direct contact with the anterior rectal wall. An additional margin was added around the PTV to account for penumbra.

RT was planned in two phases. The first was carried out with IMRT step-and-shoot with 5-fields distributed at angles of 35°, 100°, 180°, 260°, and 325° respectively. A total dose of 60 Gy in the prostate and 54 Gy in the seminal vesicles (30 fractions of 2 Gy and 1.8 Gy, respectively) was delivered in this phase. In the second phase, 7 to 10 fractions of 2 Gy, totaling 74 to 80 Gy in the prostate were delivered with 3 fields distributed at angles of 0°, 90°, and 270° planned with three-dimensional conformal RT. The prescription dose should comprise at least 95% of the PTV.

Dose constraints for the respective organs at risk were: a) rectum - V70 Gy < 20%, V60 Gy < 35%, V50 Gy < 50%; b) bladder - V80 Gy < 15%, V75 Gy < 25%, V70 Gy < 30 - 35%, V50 Gy < 55-60%; c) femoral head - V50 Gy < 5%, maximum dose < 55 Gy; and d) penile bulb - mean dose < 52.5 Gy.

### Clinical outcomes

During the course of irradiation, patients were evaluated weekly by their radiation oncologists. Clinical follow-up after treatment was performed at 3- to 5-month intervals over 5 years. Outcomes were measured from the conclusion of RT to the first event or last visit. Biochemical failure (BF) was defined according to the Houston criteria (PSA nadir + 2.0 ng/dL) [5]. Acute and late GI and GU toxicities were assessed using the National Cancer Institute Common Terminology Criteria for Adverse Events (NCI CTCAE), version 3.0^,^ which can be briefly summarized as follows: a) grade 1: minimal adverse effects with no need for medication; b) grade 2: adverse effects with symptoms that require medication; c) grade 3: adverse effects requiring small-scale procedures (e.g., cauterization, catheterization, blood transfusion, argon coagulation therapy); and d) grade 4: life-threatening risks that require large-scale surgeries and hospitalization. Acute side effects were defined as those occuring during the course of radiation or within 90 days of its completion. Late toxicity was defined as occurring after the 3^rd^ month of follow-up time.

### Statistical analysis

The studied characteristics were expressed as absolute and relative frequencies. Survival curves based on the Kaplan-Meier function were constructed with the estimates of the mean biochemical control times for each characteristic of interest. The relative risks of biochemical failure for each characteristic of interest were estimated with their respective 95% confidence intervals (CI). Mean metastasis-free and overall survival times were estimated with the Kaplan-Meier method. GU and GI toxicity were expressed as absolute and relative frequencies. Correlations between dose level and acute GU and GI toxicities and with late GU and GI toxicities were investigated with Spearman’s correlation analysis.

SPSS® v17.0 statistical package software (Chicago, Illinois) was used for the analysis with significance level set at 5% (p ≤ 0.05).

## Results

Of the 140 patients included in the study, most were white (89%). Mean age was 68.6 years (Standard Deviation = 7.9 years) and median follow-up time was 58.3 months (range: 12.3 to 138.9 months). Table 1 summarizes patients’ characteristics, including tumor stage, pre-treatment PSA value, Gleason score, risk group, and prescription dose.

**Table 1:** Characteristics of the 140 included patients

**Characteristic**		
**Age (years)**		
Median	70	
Mean	69	
Range	45–83	
**Median follow-up (months)**	58.3	
	**Number**	**%**
**Race**		
White	125	89.3
Black	5	3.6
Other	10	7.1
**T Stage**		
T1a	5	3.6
T1b	3	2.1
T1c	65	46.5
T2a	51	36.4
T2b	8	5.7
T2c	3	2.1
T3a	4	2.9
T3b	1	0.7
**Gleason score**		
≤ 6	53	38
7	56	40
8 – 10	31	22
**PSA**		
< 10	106	75.7
10 – 19.9	28	20
>20	6	4.3
**NCCN Risk Group**		
Low	36	25.7
Intermediate	64	45.7
High	40	28.6
**Prescribed dose levels (Gy)**		
74	18	13
76	20	14
78	50	36
80	52	37

### Biochemical control

**Figure 1:**
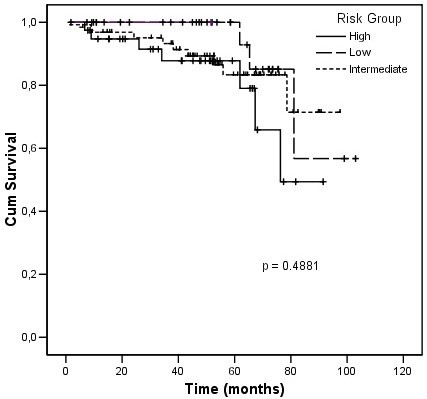
Biochemical failure free survival.

Biochemical failure occurred in 13.6% of patients. The biochemical failure-free survival rate according to the NCCN risk classification is presented in Figure 1. Actuarial 5-year biochemical control rates were 91.7%, 82.5% and 85.9% and for low-, intermediate, and high-risk patients, respectively with mean biochemical-free survival rates of 90.9%, 85.1% and 74.6% (p = 0.4881).

Table 2 shows that only clinical T stage affected biochemical control. Stage T2 patients showed a higher risk of biochemical failure than stage T1 patients (RR = 2.91; 95% CI: 1.04; 8.17). No statistically significant differences were observed in the other analyzed variables (Gleason score, PSA, risk group, and age).

**Table 2:** Relative risks for PSA relapse-free survival according to the studied variables

**Variable**	**RR**	**95% CI**	**Total**	**PSA relapse**	**%**	**p**
**Stage**						0.058
T1a/T1b/T1c	1.00		73	5	6.8	
T2a/T2b/T2c	2.91	1.04 – 8.17	62	13	21.0	
T3a/T3b	6.02	0.67 – 54.22	5	1	20.0	
**Gleason score**						0.774
≤ 6	1.00		53	6	11.3	
7	1.08	0.37 – 3.12	56	8	14.3	
8 – 10	1.50	0.46 – 4.94	31	5	16.1	
**PSA**						0.493
< 10	1.00		106	13	12.3	
10 – 19.9	1.74	0.61 – 4.97	28	5	17.9	
≥ 20	2.07	0.27 – 16.21	6	1	16.7	
**NCCN Risk Group**						0.331
Low	1.00		36	3	8.3	
Intermediate	1.61	0.43 – 5.94	64	9	14.1	
High	2.62	0.67 – 10.16	40	7	17.5	
**Age (years)**						0.324
< 60	1.00		21	2	9.5	
≥ 60 e < 75	2.15	0.48 – 9.49	86	15	17.4	
≥ 75	0.89	0.12 – 6.53	33	2	6.1	
**Total**			**140**	**19**	**13.6**	

*Abbreviations*: NCCN = National Comprehensive Cancer Network; PSA = prostate-specific antigen; RR = relative risk; CI = confidence interval.

### Metastasis-free survival and overall survival

**Figure 2:**
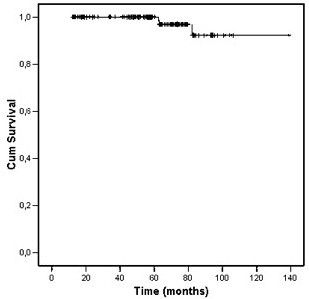
Distant metastasis free survival.

Distant metastases occurred in 3 (2%) patients. The actuarial 5-year metastasis-free survival rate was 96% (Figure 2). Five patients (4%) died and prostate cancer was the cause of death in two. The actuarial 5-year overall survival rate was 97.5%.

### Toxicity

Incidences of acute and late GI and GU toxicities are depicted in Table 3. Acute GU toxicity occurred in 82.1% of patients, while late grade ≥ 2 GU toxicity occurred in 20,2%.

**Table 3:** Gastrointestinal and genitourinary acute and late toxicity rates

**Grade**	**GI toxicity N (%)**		**GU toxicity N (%)**	
	**Acute**	**Late**	**Acute**	**Late**
**None**	53 (37.9)	97 (76.4)	24 (17.1)	85 (65.9)
**1**	45 (32.1)	10 (7.9)	31 (22.1)	18 (14)
**2**	42 (30)	16 (12.6)	84 (60)	24 (18.6)
**3**	0	4 (3.1)	1 (0.7)	2 (1.6)
**4**	0	0	0	0

*Abbreviations*: *GI* gastrointestinal, *GU* genitourinary, *N* number of patients.

Any grade of acute GI toxicity was observed in 62% of the patients. Late grade ≥ 2 GI toxicity occurred in 16%, four (3%) patients presented late grade 3 GI toxicity and were submitted to small-scale procedures. Acute GU and GI toxicities were associated with higher incidence of GU and GI late toxicities (p < 0.05), and there was a statistically significant correlation between the occurrence of acute GI and acute GU toxicity (p < 0.001).

## Discussion

This study reports the results of disease control and toxicity in patients with clinically localized prostate cancer treated with definitive IMRT alone with 1 cm posterior PTV margin and doses above 74 Gy.

Excellent disease control rates were observed, with 5-year biochemical failure-free survival of 91.7%, 82.5% 85.9% for low-, intermediate-, and high-risk patients, respectively.

A unique feature of our series is that none of the patients received any kind of HT and/or pelvic lymph node irradiation, even those classified as high or intermediate risk. All high-risk patients in this study were offered 2–3 years of HT, and all intermediate-risk patients were offered 6 months of HT. These patients did not receive HT either due to potential side effects, or comorbidities, or patient preference.

The role of HT concomitant with RT is well established for high-risk prostate cancer patients [6-9] however, most published studies used low doses (< 74 Gy) with pelvic lymph node irradiation, and focused on patients with locally advanced disease. In that specific group, HT is almost mandatory, given that it is associated with higher rates of overall survival.

In intermediate- and high-risk patients with localized, small-volume disease, i.e., those with a Gleason score of ≥ 7 and/or PSA ≥ 10 ng/ml and ≤ T2c, the benefits of HT are not very clear. In our series, 99 patients presented intermediate- or high-risk prostate cancer with localized, small-volume disease. Of these, 15 (15%) had biochemical failure, 3 (3%) presented metastases, and 1 (1%) died of prostate cancer in a median follow-up period of 58 months. Stage T2 patients presented a risk of biochemical failure almost three times higher than the stage T1 patients. In a study reported by D’Amico et al., intermediate- and high-risk stage ≤ T2b prostate cancer patients were randomized into groups that received either RT together with 6 months of HT or RT only (both treatments with a dose of 70 Gy). The group that received combined treatment presented a 21% rate of biochemical failure and no death from prostate cancer in a median follow-up period of 54 months [10]. Our results suggest that the additional benefit of HT could be replaced by RT doses above 74 Gy, as the role of radiation dose escalation is well documented to increase biochemical control and reduce clinical failure [1,11]. Because HT is associated with some significant side effects [12], replacing it with high-dose RT, as demonstrated in our series, could represent a less toxic treatment strategy for these patients. Yet, no randomized studies have tested this hypothesis. Once completed, the Radiation Therapy Oncology Group (RTOG) 0815 protocol may help to answer this question.

Despite the benefits offered by dose escalation, higher toxicity rates are expected. Series that used doses above 74 Gy and 3D-RT showed rates of late grade ≥ 2 GI and GU complications of 16–35% and 15–37%, respectively [13-17]. Series in which IMRT was used with doses above 74 Gy, rates of late grade ≥ 2 GI and GU complications were 1.5–24% and 5–29%, respectively [3,18,19]. A comparison of complication rates associated with 3D-RT and IMRT strongly suggests that rates are lower for IMRT [20,21].

In our study, rates of late grade 2 GI and GU complications were 12.6% and 18.6%, respectively, and comparable values for grade 3 were 3.1% and 1.6%. Tools for measuring the degree of toxicity are not standardized, and the assessment methods remain subjective. Though, there is striking variation in complication rates between the different published series. For example, in the Alicikus et al. study, 170 patients with prostate cancer received 81 Gy using IMRT and late grade 2 GI toxicity occurred in 4 patients (2%). Among these patients, 2 developed grade 2 rectal bleeding [3]. In our study, 16 patients developed grade 2 GI toxicity but only 3 developed grade 2 rectal bleeding.

Another finding of our study was a significant correlation between acute and late toxicities, as between acute GI and GU toxicities. Similarly, in a study by Zelefsky et al., the authors detected that the occurrence of acute grade ≥ 2 GI and GU toxicity was significant predictor of late grade ≥ 2 GI and GU toxicity [21]. These findings may help to identify those patients who should be followed closely for late side effects.

In the current study, a symmetric PTV margin of 1 cm around the CTV (including the posterior margin) was used. There is no consensus on the ideal safety margin to use, which is typically determined by technical considerations at each treatment facility. However, usually the posterior margin is reduced in order to achieve a better rectal sparing, despite the risk of missing the target. Table 4 shows an overview of late toxicities in some IMRT dose-escalation reports and the posterior margin used. Even with larger margin, our results were comparable with those with smaller margins.

**Table 4:** Overview of late toxicities reports using high dose IMRT

**Study**	**N**	**Posterior margin (cm)**	**Mean dose (Gy)**	**GI toxicity (%)**		**GU toxicity (%)**	
				**G2**	**G3**	**G2**	**G3**
Current study	140	1	78	13	3	18	2
Alicikus et al [3]	170	0.6	81	2	1	9	5
Jani et al [18]	106	0.6	76	3	3	19	4
De Meerleer et al [19]	133	0.7	76	17	1	19	3

*Abbreviations:N* number of patients, *GI* gastrointestinal, *GU* genitourinary, *G* grade.

A major concern regarding the use of IMRT in treating prostate cancer is the risk of missing the target due to the internal movement of organs. Some published studies have suggested that the inter and intrafractions changes in rectal volume could compromise effective treatment [22-24]. This risk is directly related to the PTV margins definition which is related to the precision of each technique.

Image-guided radiotherapy (IGRT) is the best method to safely reduce the margins, leading to a reduction of complication rates without compromising disease control. Zelefsky et al. recently reported the toxicity rates and biochemical control outcomes in patients treated with high dose IMRT using IGRT for clinically localized prostate cancer. One hundred eighty-six patients were treated at a dose of 86.4 Gy with daily correction of the target position based on kilovoltage imaging of implanted prostatic fiducial markers. This group of patients was retrospectively compared with a similar cohort of 190 patients who were treated with IMRT to the same prescription dose but without implanted fiducial markers in place (non-IGRT). The 3-year likelihood of grade 2 and higher urinary toxicity for the IGRT and non-IGRT cohorts were 10.4% and 20.0%, respectively (p = 0.02) [25].

In our study, no image-guidance was used, and the excellent rates of local control, even with no HT, are probably due to the high doses and PTV margins used. And, as already pointed, unlike to what would be expected, toxicity rates were low, and comparable to the already published ones.

In 2006 we started IGRT at our department with cone beam CT for some patients receiving IMRT for prostate cancer. In the first moment we decided to keep the same margins of 1 cm in all directions. After 2009 we formally initiated to delivery radiation for these patients with smaller margins (0.5 to 0.7 cm) and daily cone beam CT. We expect that the use of IGRT will be related with less morbidity, given its higher precision.

The major limitations of the current study, including the biases inherent to a retrospective review, are the physician-based assignment of toxicities scores which warrant consideration in interpretation of the results, the low number of patients studied and the short follow-up. A longer follow-up time is needed to determine the duration of tumor control and to assess the incidence of additional late complications.

## Conclusions

Some patients with clinically localized prostate cancer may benefit from RT as a single treatment modality. The IMRT technique used in this cohort, delivering doses of 74 Gy or higher with 1 cm posterior PTV margin was safe, well-tolerated, and effective.

## Abbreviations

RT: Radiotherapy; GI: Gastrointestinal; GU: Genitourinary; 3D-RT: Three-dimensional conformal radiotherapy; IMRT: Intensity-modulated radiotherapy; PTV: Planning target volume; HT: Hormone therapy; NCCN: National comprehensive cancer network guidelines; CTV: Clinical target volume; BF: Biochemical failure; NCI CTCAE: National Cancer Institute Common Terminology Criteria for Adverse Events; CI: Confidence intervals; RTOG: Radiation Therapy Oncology Group; IGRT: Image-guided radiotherapy.

## Competing interests

The authors declare that they have no competing interests.

## Authors’ contributions

RG carried out the data collection and drafted the manuscript. ETL and FGG participated in the data collection. GND, FFA, CVA, SAH, JFS, HAC and BG provided comments, critique and suggestions for its improvement. CKH carried out radiotherapy planning and provided comments, critique and suggestions for its improvement. All authors read and approved the final manuscript.
